# The influence of a biopsychosocial educational internet-based intervention on pain, dysfunction, quality of life, and pain cognition in chronic low back pain patients in primary care: a mixed methods approach

**DOI:** 10.1186/s12911-015-0220-0

**Published:** 2015-11-23

**Authors:** Fran Valenzuela-Pascual, Fidel Molina, Francisco Corbi, Joan Blanco-Blanco, Rosa M. Gil, Jorge Soler-Gonzalez

**Affiliations:** 1 Universidad de Lleida, Facultad de Enfermería y Fisioterapia, Montserrat Roig 2, Lleida, 25198 Spain; 2 Grupo de Estudios Sociedad, Salud, Educación y Cultura, Universidad de Lleida, Pl. de Víctor Siurana 1, Lleida, 25003 Spain; 3 Grup de Recerca de Cures de Salut, Institut de Recerca Biomèdica, Avda Alcalde Rovira Roure 80, Lleida, 25198 Spain; 4 Universidad de Lleida, Facultad de Educación, Psicología y Trabajo Social, Av. Estudi General 4, Lleida, 25001 Spain; 5 Universidad de Lleida, Institut Nacional d’Educació Física de Catalunya, Centro de Lleida, Partida Caparrella s/n, Lleida, 25192 Spain; 6 Departamento de Informática e Ingeniería Industrial, Universidad de Lleida, Jaume II 69, Lleida, 25001 Spain; 7 Universidad de Lleida, Facultad de Medicina, Montserrat Roig 2, Lleida, 25198 Spain; 8 Institut Català de la Salut, Rambla de Ferran 44, Lleida, 25007 Spain

**Keywords:** Low back pain, Patient education, Educational technology, Pain neurophysiology, Gamification

## Abstract

**Background:**

Low back pain is the highest reported musculoskeletal problem worldwide. Up to 90 % of patients with low back pain have no clear explanation for the source and origin of their pain. These individuals commonly receive a diagnosis of non-specific low back pain.

Patient education is a way to provide information and advice aimed at changing patients’ cognition and knowledge about their chronic state through the reduction of fear of anticipatory outcomes and the resumption of normal activities. Information technology and the expedited communication processes associated with this technology can be used to deliver health care information to patients. Hence, this technology and its ability to deliver life-changing information has grown as a powerful and alternative health promotion tool.

Several studies have demonstrated that websites can change and improve chronic patients’ knowledge and have a positive impact on patients’ attitudes and behaviors. The aim of this project is to identify chronic low back pain patients’ beliefs about the origin and meaning of pain to develop a web-based educational tool using different educational formats and gamification techniques.

**Methods/design:**

This study has a mixed-method sequential exploratory design. The participants are chronic low back pain patients between 18–65 years of age who are attending a primary care setting. For the qualitative phase, subjects will be contacted by their family physician and invited to participate in a personal semi-structured interview. The quantitative phase will be a randomized controlled trial. Subjects will be randomly allocated using a simple random sample technique. The intervention group will be provided access to the web site where they will find information related to their chronic low back pain. This information will be provided in different formats. All of this material will be based on the information obtained in the qualitative phase. The control group will follow conventional treatment provided by their family physician.

**Discussion:**

The main outcome of this project is to identify chronic low back pain patients’ beliefs about the origin and meaning of pain to develop a web-based educational tool using different educational formats and gamification techniques.

**Trial registration:**

ClinicalTrials.gov NCT02369120 Date: 02/20/2015.

**Electronic supplementary material:**

The online version of this article (doi:10.1186/s12911-015-0220-0) contains supplementary material, which is available to authorized users.

## Background

Low back pain (LBP) is the most commonly reported musculoskeletal problem in the world [1–7]. Up to 90 % of these individuals have no clear explanation for the source and origin of their pain and are diagnosed with non-specific LBP. Although LBP is recurrent, a large number of treatments addressing this problem have modest clinical benefits and low adherence [8, 9].

Pain is a multifactorial experience associated with psychological and emotional factors that play important roles in the transition from the acute to chronic states [10–12]. Additionally, there is evidence that disability in chronic low back pain (CLBP) patients is caused by fear of injury and pain avoidance and that these chronic patients have high levels of fear-avoidance beliefs [13, 14]. Studies have shown that patients suffering from CLBP have an altered interpretation of their pain based on their assumption of further tissue damage and catastrophic consequences rather than evidence [15, 16].

Compared with patients with acute LBP whose levels of fear-avoidance beliefs (FAB) decrease over time through the healing process, CLBP patients exhibit persistent elevated levels of FABs that remain unchanged [13, 17, 18].

Patient education could be a way to provide information and advice aimed at changing patients’ cognition and knowledge about their chronic state to reduce fear of serious outcomes and allow the resumption of normal activities [19–21]. The use of pain neurophysiology as an educational intervention (PNI) has been proven to be effective in pain-related problems other than CLBP [22–24]. Other types of patient education include interventions specifically focused on ergonomy and exercises based on anatomic and biomechanical models. In contrast, the neurophysiology of pain as a type of educational intervention for chronic pain patients is focused on describing the mechanisms of peripheral and central processing of the nociceptive signal and explaining how this transmission is modulated by brain processing and influenced by psychosocial factors. Thus, patients learn that the meaning of their pain is not always related to the tissue damage of painful structures. Furthermore, this type of education helps patients reconceptualize their pain, resulting in better functional outcomes and reducing disability and catastrophic thoughts [24].

The use of information and communication technology in health care to deliver information to patients is becoming a powerful tool. The internet is an example of a new technology that is part of our everyday lives. Thanks to the wide spectrum of possibilities that the internet offers, different authors have proposed using it as an important platform to display high-quality interactive evidence-based information [25–30]. Other studies have demonstrated that websites can change and improve chronic patients’ knowledge. Importantly, websites can have a positive impact on patients’ attitudes and behavior [25, 31–36], especially for physical and mental health problems [26, 37–40].

### Rationale of the study and study hypothesis

Nine studies [41–49] examining the effect of PNI on physical performance, pain cognition and disability in CLBP reported positive effects. However, there was a lack of standardization of the type of information provided. Seven studies provided detailed information concerning the PNI [41, 43, 45–49], whereas only five studies specifically addressed the description of the different components of the PNI [43, 45–47, 49]. Although promising, there is no strong evidence for the use of PNI for CLBP patients because this evidence has been rated as very low quality by some authors due to the lack of good randomized controlled trials [19, 50]. Moreover, the authors in all of the studies developed the PNI without exploring the needs of CLBP patients. Therefore, the rationale of this protocol is to develop an educational tool focused on of beliefs and knowledge of patients towards their CLBP. This rationale justifies the use of a mixed methodology wherein the authors explore the patients’ beliefs and knowledge about their pain for the subsequent development of an educational patient-centered tool. The authors understand that an educative tool must take into account the patients’ thoughts and beliefs.

Therefore, the main outcome of this project is to identify chronic low back pain patients’ beliefs concerning the origin and meaning of their pain to develop a web-based educational tool using different educational formats and gamification techniques.

The hypotheses being tested by our project are:

- Persistent chronic low back pain and its consequences are maintained by the lack of understanding of the origin and meaning of pain.
- Pain neurophysiology as an educational internet-based intervention for chronic low back pain will change cognition about the origin and meaning of pain, with the outcome of pain reduction, disability reduction, and better quality of life compared to normal care.

There are specific objectives for each phase of the study.

Phase 1 (QUAL):

- To identify chronic low back pain patients’ beliefs concerning the origin and meaning of pain.

Phase 2 (Connecting procedure):

- To construct and develop a biopsychosocial web-based educational intervention using the QUAL results.

Phase 2 (QUAN):

- Primary outcome. To evaluate the effect of a biopsychosocial web-based educational intervention for chronic low back pain based on pain intensity compared to normal care.
- Secondary outcomes. To assess the effect of a biopsychosocial web-based educational intervention for chronic low back pain compared to normal care on:
  - oFear avoidance beliefs.
  - oKinesiophobia.
  - oPain catastrophizing.
  - oDisability.
  - oQuality of life.

One of the utilities of QUAL is to better understand the beliefs of chronic low back pain patients concerning their pain, including the origin and meaning of their pain. This information is paramount not only to aid in the construction of the web site but also for health professionals involved in the diagnosis and treatment of these patients.

If the web-based intervention is found to be successful in modifying CLBP patients’ pain cognition, this will open the possibility of including this web site-based approach in future chronic pain management programs in primary care. Moreover, the web site will be developed with the future aim of including and adding new technologies to help patients and professionals involved in chronic pain.

## Methods/design

### Ethical issues

The study follows the Declaration of Helsinki and the “Guidelines for Good Clinical Practice” (CPMP/ICH/135/95) and has been approved by the Ethical Committee of Clinical Research in Primary Care IDIAP in Catalonia, Spain (P14/138).

Because our intervention does not involve any physical activity/intervention, we do not expect to have any physical side effects. Patients in the experimental group will be advised to contact their family physician if they experience any physical problems or worsening of their condition. Additionally, all subjects in the experimental group will be able to contact the researchers through the web site.

### Study design

To answer the research question, the authors will use a mixed-method sequential exploratory design. The purpose of mixed methodology is not to replace qualitative or quantitative methodologies, but to use the strengths of both while reducing their weaknesses [51]. Specifically, the sequential exploratory design includes an initial qualitative phase followed by a quantitative phase, with the aim of developing an educational tool [52, 53]. In this project, we propose that both phases (qualitative and quantitative) must have the same relevance (QUAL-QUAN) for the development of the educational tool and that the development of the study must be conducted in three stages [54]:

1. Qualitative data collection through semi-structured personal interviews followed by thematic analysis.
2. Construction of the educational tool with the results obtained in the previous step (topics or emerging categories).
3. Analysis of the effectiveness of the educational tool using a randomized controlled trial.

The use of a mixed-method design is fully justified in this protocol because the integration of both methodologies (QUAL-QUAN) occurs when the data from the qualitative phase contribute to the construction of the educational tool [53].

### Subjects

The recruitment process will be performed independently in each phase of the study, although in both phases this recruitment process will take place in the same primary care centers in the city of Lleida through family physicians. The inclusion and exclusion criteria are also common to both phases. Prior to the beginning of the first phase, the first author of this study will perform a presentation of the project to the medical and nursing staff in each of the primary care centers involved in the study to ask for their cooperation.

QUAL: In this phase, patients will be recruited by their respective family physicians. Once the physician makes the diagnosis of CLBP and ensures that the individual meets the inclusion and exclusion criteria, the physician informs the patient about the existence of this project and invites him to contact the first author by telephone. If the person agrees to participate in the study, the interview will be scheduled to take place in the Faculty of Nursing and Physiotherapy of the University of Lleida.

QUAN: The recruitment process will start after the end of QUAL and the development of the educational tool. This phase will consist of an educational intervention using a randomized controlled trial design. The recruitment process will be the same as in QUAL. Prior to the beginning of the intervention, the first author will meet the study subjects individually to inform them of the study conditions and provide them with the informed consent form.

### Inclusion criteria

- History of CLBP longer than 6 months.
- Patients between 18–65 years of age.
- Able to read, speak and understand Spanish and Catalan.
- Access to the Internet, a computer or laptop and e-mail address.
- Accept and sign the informed consent form.

### Exclusion criteria

Any red flag condition [55]:

- Onset age < 20 or > 55 years.
- Non-mechanical pain (unrelated to time or activity).
- Thoracic pain.
- Previous history of carcinoma, steroid use, or HIV infection.
- Feeling unwell.
- Weight loss.
- Widespread neurological symptoms.
- Structural spinal deformity.

### Sample size

QUAL: In this phase, a purposive convenience sample will be used to achieve a representative number of patients with CLBP [56]. To ensure the discursive significance of our results, the sample will include an equal number of subjects from different age groups (18–29 and 30–65), genders, and educational levels.

QUAN: The sample size calculation is based on the study of Ryan et al. [48] and the systematic review of Clarke et al. [50]. From these studies and taking into account other similar studies such as Ostelo and colleagues [57] and Furlan et al. [58], we assume that pain measurement in the patients at baseline will be approximately 50 ± 18 on a scale of 0 to 100. We assume that a reduction of 30 % (15 points) in the pain scale will be sufficient to be considered clinically relevant.

Accepting an alpha risk of 0.05 and a beta risk of 0.2 (statistical power of 80 %), using a two-sided test, and assuming a 20 % withdrawal rate, it will be necessary to include 29 subjects in each group to detect a difference greater than or equal to 15 units (assuming a baseline distribution of 50 ± 18). We assume that there will be a 20 % withdrawal rate.

The researchers will use a simple randomization technique. An external researcher from the Higher Polytechnic School of the University of Lleida will generate the randomization assignment using the STATS® program [59] and keep the assignments on a computer assigned for this study that is inaccessible to the rest of the staff.

### Study variables

Pain intensity:

**-**Visual Analog Scale (VAS): This scale was developed by Huskinson in 1976 [60] as a method to measure pain intensity. VAS is an easy, simple and reproducible tool that can be used by the same patient on multiple occasions. The scale consists of a 10-cm line with a description on both extremes. “No pain” is on the far left and “worst pain ever” is on the far right of the scale. For some authors, VAS is the most sensitive measurement in clinical pain research [60–62].
- Cognition:
**-** Fear Avoidance Beliefs Questionnaire (FABQ): The FABQ is a self-reported questionnaire that consists of 16 items about the beliefs of LBP patients on the influence that physical activity and work have on their pain. Each item can be scored from 0 (totally agree) to 6 (totally disagree) [63, 64]. The Spanish version of the FABQ has demonstrated good reliability [65].
**-** Tampa Scale for Kinesiophobia (TSK): This scale is widely used in pain medicine to assess pain-related fear. The Spanish format has 11 items, with each score ranging from 1 (totally disagree) to 4 (totally agree). The Spanish version of the TSK is easy to use, reliable and valid [64].
**-** Pain Catastrophizing Scale (PCS): The PCS was developed in 1995 by Sullivan and colleagues [66] to facilitate research on how catastrophic thinking impacted the patients’ pain experiences. This is a self-administered scale of 13 items that evaluates different aspects of the pain experience, such as rumination, magnification, and helplessness. The PCS uses a 5-point Likert scale to evaluate the items with the end points of 0 (not at all) and 4 (all the time); a score above 30 indicates a clinically relevant level of catastrophizing [66]. This scale has been shown to be adequate in terms of excellent internal consistency in different studies, including Spanish populations [66–69].

Disability:

**-** Roland-Morris Questionnaire (RMQ): This is a self-reported questionnaire assessing function and disability. It is an easy instrument for patients with scores ranging from 0 to 24; a change of 4 or more points is considered clinically important [48, 70]. The RMQ is reliable, valid, and adequate to assess disability in patients with LBP [48, 70, 71], and the Spanish version has been successfully validated [71].

Quality of life:

**-** Health Survey SF-36: This questionnaire was developed in 1992 by Ware and Sherbourne [72] to assess the health status in clinical practice and research in the Medical Outcomes Study. It consists of 36 items covering 8 health concepts: physical function, bodily pain, social functioning, vitality, mental health, general health, emotional health, and physical activity. The SF-36 has been used in people over 14 years of age. A high score represents a better quality of life. This questionnaire has been used in multiple studies with different health conditions [73], including chronic low back pain patients [74], and has been validated in Spanish [75].

Other variables:

- Other variables will also be measured, including acceptability, patient satisfaction, and the number of times that the information provided in the web site is watched/used by subjects in the experimental group.

### Web site development and educational material

Staff of the Higher Polytechnic School of the University of Lleida will assist in the development of the web site and the educational material. Once the results of QUAL have been obtained, the authors will generate a variety of topics related to patients’ misbeliefs about their CLBP. The outcome of the educational material is to answer wrong pain conceptions using the neurophysiology of pain as a starting point. This educational material will be evidence-based and elaborated using understandable language and different formats, including video and 2-3D animation. Therefore, the platform will provide patients with customized tasks that allow them to use the metaphor of the journey (the narrative as a dynamic of the game) to feel that they manage their own path, which will change negative perceptions into positive perceptions about certain actions. At all times, the patients will be able to choose among different information sources, such as videos about the origin of chronic pain, 3D representations of different neurophysiological processes, and FAQs. Additionally, the patients will be able to contact a specialist in the neurophysiology of pain by email or videoconference. Furthermore, to reinforce patients’ motivation and participation, gamification techniques (defined as elements forming part of the design of video games but used in a different context) will be implemented [76–78].

The web site will be developed using Drupal as a content management system. Some modules of the management system, including those related to questionnaires and video tutorials, will be modified and adapted to the needs of the project. This will increase the versatility of the platform, resulting in better utilization of the majority of the systems (i.e., the registration modules by adding security for the available data using Advanced Encryption Standard, synchronization, and mass mailing modules).

### Data collection

Data collection will be conducted in two phases (QUAL and QUAN).

QUAL: To meet the qualitative objective of this study, the authors will use semi-structured personal interviews because they are very useful for obtaining a better and wider understanding of the issues related to the chronic problem from the patient's perspective [79–81]. The interviews will be conducted individually by the first author of the study in Spanish or Catalan (the mother tongue of the interviewer and interviewees), and digitally recorded with the interviewee’s informed written consent. Interviews will take place in the Faculty of Nursing and Physiotherapy of the University of Lleida to generate a neutral and comfortable setting different from the primary care consultation [82]. The interviewer will use an interview guide (see Additional file 1) produced after reviewing the literature and based on the experience and knowledge of the research team. Interviews will be analyzed by the first author using inductive thematic analysis to identify common patterns across the interviews and finally generate potential themes to use in the development of the educational tool [83, 84]. To ensure the rigor and reliability of the material, an independent coder will also analyze the interviews [84], and all of the authors will be involved in the last step of interpreting the data and constructing the web-based educational tool.

QUAN: The intervention will last for two weeks, and variables will be measured pre- and post-test. The researchers based the duration of the intervention on the study of Keulers et al. [85], which had similar characteristics. An external researcher from the Higher Polytechnic School of the University of Lleida will be responsible for the maintenance of the website. Moreover, this external researcher will introduce the data from the subjects and the questionnaires used in this study. The first author of this study will meet with the study subjects to inform them about the study and deliver the informed consent. Once individuals have signed the consent form, they will be given a password to access the web platform at any time from any computer or laptop until the end of the trial. After logging into the website, patients will be asked to fill in the questionnaires. Once this step is completed, subjects from the control group will see a message on the screen asking them to follow the conventional treatment provided by their family physician in primary care, and they will be reminded that in two weeks they must re-access the website to again fill in the questionnaires. This treatment is based on the clinical guidelines of the Catalan Institute of Health. Subjects from the experimental group will see a different message requesting them to access the “educational tool” on the homepage of the website. Only subjects from the experimental group will have access to the educational tool using their personal password. Both groups of subjects will receive two email messages during the study (one two days before the end of the study and the second one the last day) to remind them to fill out the questionnaires again.

### Statistical analysis

QUAN: Quantitative variables will be described using the mean, standard deviation, median and interquartile range. The bivariate analysis will be contrasted using the Student’s *t*-test or Chi-square test or a non-parametric test if a normal distribution cannot be assumed. Pain is the main numeric variable, with a range between 0–100. The differences between groups will be contrasted using the Student’s *t*-test or the Mann–Whitney test when assuming a non-normal distribution. The statistical analysis will be performed with an alpha of 0.05 using the SPSS and R software.

### Possible limitations

One limitation in QUAL is that a single interviewer will perform all interviews and one analyst will perform the initial thematic analysis. Although both the interview and analytic processes will be validated by an external researcher, this may be a source of potential bias. Another limitation may come from the author’s interpretation of the data provided by the subjects of the study; this potential bias will be overcome using triangulation of researchers. The researcher’s perspective can be accepted as rigorous if appropriate tools for appraising quality are used [86, 87]. Moreover, the purpose of this study is not to provide accurate data on CLBP but to contextualize the patients’ perception within their own experience [88, 89] to construct a web-based educational tool.

One of the possible limitations in QUAN is that participants will know which group they are allocated into, making the study blinded only to the researchers. The sample size may represent another limitation. A smaller than expected final sample size increases the likelihood of a type II error and limits the internal validity of the study, leading to inconclusive results.

Regardless, our results will be limited to CLBP patients and will need to be replicated in randomized control trials with a larger sample size.

Finally, there is another possible limitation derived from the use of new technologies. In our project, the web-based intervention will contain educational materials in different formats, such as video, audio, and 3D images. However, as noted by Camerini et al. [90], other alternatives or different formats may be better accepted by subjects in the experimental group, thereby improving their experience.

## Discussion

This study will use mixed methods to identify chronic low back pain patients´ beliefs about the origin and meaning of pain to develop a web-based educational tool using different educational formats and gamification techniques. Stage one of the study will identify chronic low back pain patients´ beliefs concerning the origin and meaning of pain using qualitative data collection through semi-structured personal interviews. Stage two of the study will allow the construction of the educational tool with the results obtained in the previous stage (topics or emerging categories). Stage three of the study will analyze the effectiveness of the educational tool using a randomized controlled trial.

Our investigation will open the possibility of including this web site-based approach in future chronic pain management programs in primary care. Moreover, the web site will be developed with the future aim of including and adding new technologies to help patients and professionals involved in chronic pain.

## Additional file

Additional file 1: **Interview guide.** (PDF 241 kb)

## Abbreviations

LBP: Low back pain
CLBP: Chronic low back pain
FAB: Fear-avoidance beliefs
PNI: Pain neurophysiology as an educational intervention
P1: Phase 1
P2: Phase 2
IDIAP: Clinical Research in Primary Care
VAS: Visual Analog Scale
FABQ: Fear Avoidance Beliefs Questionnaire
TSK: Tampa Scale for Kinesiophobia
PCS: Pain Catastrophizing Scale
RMQ: Roland-Morris Questionnaire
