# Supplementary material for: The influence of a biopsychosocial educational internet-based intervention on pain, dysfunction, quality of life, and pain cognition in chronic low back pain patients in primary care: a mixed methods approach
Source: BMC Med Inform Decis Mak. 2015 Nov 23;15:97. doi: 10.1186/s12911-015-0220-0 (PMC4657202; doi:10.1186/s12911-015-0220-0)
Supplement: Additional file 1: — Interview guide. (PDF 241 kb) [file 12911_2015_220_MOESM1_ESM.pdf]

Interview guide

1	For how long have you had this low back pain?
2	Do you remember how did this pain begin?
3	What do you do every time you feel the pain?
4	Using your own words, could you tell me what have you been told about your pain?
5	What kind of treatments have you had?
6	Even though you have had different treatments, why do you think the pain is still there?
7	What does this pain mean for you? In other words, when it hurts, why do you think it hurts?
8	Do you think the health professionals who have treated you have understood your pain?
9	What activities or hobbies have you stopped doing since this low back pain started?
10	Why?
11	In reference to your back pain, if you could go back in time, what things would you change? What things would you do differently?
12	In what way your pain has affected your family, work, and social life?
13	What would you like to know about your pain?
14	If you were with the world expert on chronic low back pain, what would you ask him?
